# Study of Early Elevated Gas6 Plasma Level as a Predictor of Mortality in a Prospective Cohort of Patients with Sepsis

**DOI:** 10.1371/journal.pone.0163542

**Published:** 2016-10-27

**Authors:** Grégoire Stalder, Yok Ai Que, Sara Calzavarini, Laurent Burnier, Christophe Kosinski, Pierluigi Ballabeni, Thierry Roger, Thierry Calandra, Michel A. Duchosal, Lucas Liaudet, Philippe Eggimann, Anne Angelillo-Scherrer

**Affiliations:** 1 Service and Central Laboratory of Hematology, Centre Hospitalier Universitaire Vaudois and University of Lausanne, Lausanne, Switzerland; 2 Service of Adult Intensive Care Medicine, Centre Hospitalier Universitaire Vaudois and University of Lausanne, Lausanne, Switzerland; 3 Department of Intensive Care Medicine, Inselspital, Bern University Hospital, University of Bern, Bern, Switzerland; 4 Department of Hematology and Central Hematology Laboratory, Inselspital, Bern University Hospital, University of Bern, Bern, Switzerland; 5 Department of Clinical Research, University of Bern, Bern, Switzerland; 6 Clinical Research Centre, Centre Hospitalier Universitaire Vaudois and University of Lausanne, Lausanne, Switzerland; 7 Infectious Diseases Service, Centre Hospitalier Universitaire Vaudois and University of Lausanne, Lausanne, Switzerland; Institut d'Investigacions Biomediques de Barcelona, SPAIN

## Abstract

**Background:**

Growth arrest-specific gene 6 (Gas6), a vitamin K-dependent protein interacting with anionic phospholipids and TAM tyrosine kinase receptors, is elevated in plasma of septic patients. Previous studies did not find different levels between survivors and non-survivors at admission because either they included a low number of patients (<50) or a low number of non-survivors (5%).

**Objectives:**

To determine, in a larger cohort of septic patients comprising an expected number of non-survivors, the performance of the plasma level of Gas6 and its soluble receptor Axl (sAxl) within 24 hours of admission to predict in-ICU mortality.

**Patients:**

Septic adults with or without shock.

**Methods:**

Gas6 and sAxl were prospectively measured by ELISA at day 0, 3, 7, and then weekly until discharge or death.

**Results:**

We evaluated 129 septic patients, including 82 with and 47 without shock, with in-ICU mortality rate of 19.4% and in-hospital mortality rate of 26%. Gas6 level was higher in non-survivors than in survivors (238 vs. 167%, *P* = 0.003); this difference remained constant during the ICU stay. The area under the ROC curve for Gas6 (0.695 [95% CI: 0.58–0.81]) was higher than for sAxl, procalcitonin, CRP, IL-1beta, IL-6 and-alpha, and slightly higher than for IL-8, IL-10, SOFA and APACHEII scores in predicting in-ICU mortality. Considering 249% as a cut-off value, Gas6 measurement had a negative predictive value for mortality of 87%.

**Conclusion:**

It seems that Gas6 plasma level within 24 hours of ICU admission may predicts in-ICU mortality in patients with sepsis. If our result are confirmed in external validation, Gas6 plasma level measurement could contribute to the identification of patients who may benefit most from more aggressive management.

## Introduction

Sepsis is the leading cause of mortality in the intensive care unit (ICU) [1]. Despite significant improvement in clinical management, mortality rate still reaches almost 30% [2]. These last years, important efforts have been performed to identify and describe biomarkers that could be used to assist physicians in risk stratification and decision-making processes [3]. For example, the level of blood C-reactive protein (CRP), cytokines, procalcitonin (PCT) and lipopolysaccharide (LPS)-binding protein are elevated in sepsis. However, insufficient sensitivity and specificity of these measurements prevent currently their use for early diagnosis of sepsis and management [3–5]. We recently confirmed the prognostic value of pancreatic stone protein to predict, in combination with severity scores, the outcome of patients with sepsis requiring ICU management [6,7]. However, it is necessary to identify novel sepsis biomarkers [5,8].

Growth arrest-specific gene 6 (*GAS6*) was first described in fibroblast during growth arrest [9]. It encodes Gas6, a secreted vitamin K-dependent protein expressed mainly in endothelial [10], vascular smooth muscle [11], bone marrow [12] and central nervous system cells [13]. Gas6 amino acid sequence shares 44% homology with that of protein S (PS) [10], a coagulation regulatory protein which acts as cofactor for both activated protein C and tissue factor pathway inhibitor [14, 15]. Gas6 is composed of four domains: a Gla domain, which requires gamma-carboxylation to be functional and interacts with membrane phospholipids, four EGF-like domains and a sex-hormone-binding globulin domain which comprises the binding site for receptors of the TAM (Tyro3, Axl and Mer) family [16]. TAM are tyrosine kinase receptors exhibiting a widespread expression distribution, including immune cells such as macrophages and dendritic cells [17]. TAM activation participates in a number of pathophysiological events linked to sepsis like phagocytosis, cytokine production and antigen-presentation [18,19].

Gas6 and its receptors are regulators of innate immunity, promoting anti-inflammatory responses through inhibition of cytokine production by antigen-presenting cells [20]. Gas6 is implicated in coagulation and platelets function, participating to platelet adhesion to endothelium as well as to thrombus stabilization [21–24]. Apoptotic cell phagocytosis is predominantly driven via Mer in a homeostatic environment and via Axl in an inflammatory environment [25]. Exposure of macrophages to LPS promotes the cleavage of Axl and Mer into soluble forms, which interact with Gas6 and diminish the effectiveness of apoptotic cell phagocytosis [26,27]. Toll-like Receptor activation reduces the production of Gas6 and PS which in turn facilitates pro-inflammatory cytokine production by macrophages [28]. Recent studies indicate that Gas6 influences host responses to endotoxemia and bacterial infection by modulating innate immunity and may have thereby a protective role during sepsis [20]. Furthermore, the administration of Gas6 to wild type mice with cecal ligation and puncture reduces circulating levels of IL-6 and IL-17 [29].

Plasma Gas6 levels are elevated in humans with inflammatory conditions, including sepsis [30] and correlate with organ dysfunction and disease severity [31]. Yet Gas6 plasma level at admission did not discriminate between survivors and non-survivors [30–33]. However, it should be emphasized that these studies included either few septic patients (<50) [31–33] or few non-survivors (5%) [30].

Here, in a cohort of 129 septic patients with an in-hospital mortality ranging from 20.5 to 37% [6,7] as expected in Europe [34], we investigated prospectively the performance of Gas6 plasma level at admission to predict the risk of mortality.

## Materials and Methods

### Patients

The study was performed between February 2008 and July 2012 in a 32-bed adult medico-surgical ICU of a community and referral university hospital. Patients who were at least 18 years old were evaluated within 24 hours of ICU admission for sepsis with or without shock. Infections, sepsis and septic shock were defined according to commonly used criteria [35]. As Gas6 is a modulator of innate immunity, exclusion criteria were HIV-positive status, hematological malignancies, immunosuppressive treatment and agranulocytosis. Owing to organizational constraints, inclusion was prospective but could not be strictly consecutive. Patients were followed until death or discharged from the ICU. In-ICU mortality was the primary endpoint.

The study was approved by the Institutional Review Board (Commission cantonale [VD] d’éthique de la recherche sur l’être humain, Lausanne, Switzerland). Written informed consent was obtained from patients or relatives.

### Data collection and blood sampling

Data collection was performed as previously described [6,7]. Severity of the illness was evaluated on the first day in the ICU by using the Acute Physiology and Chronic Health Evaluation II (APACHE II) score [36]. Organ dysfunction was evaluated by the Sequential Organ Failure Assessment (SOFA) score [37]. McCabe classification was recorded for each patient [38].

Citrated blood was collected at admission (Day 0), Day 3, Day 7 and then weekly until discharge from ICU or death and plasma samples were stored at -80°C until use.

### Gas6 and sAxl ELISA

To measure Gas6, we used the ELISA method developed by Clauser et al [39] with some modifications as reported below in order to globally reduce the amount of antibodies. Sensibility and specificity of the modified method were however comparable to those of the original method [39]. Wells from 96-wells plates (Maxisorp, Nunc) were coated with 50 μL per well of 5 μg/mL polyclonal goat anti-human Gas6 antibody (AB885, R&D Systems, Abingdon, United Kingdom) diluted in 0.1M NaHCO3 pH 8.2 and incubated overnight at 4°C. After two washes with PBS-Tween 0.05%, 200 μL PBS-BSA 1% were added to the wells and plates were incubated 2 hours at room temperature. After three washes, samples and normal plasma serial dilution with PBS-BSA 1% were added to the wells, followed by two hours incubation at RT. After three washes, 50 μL of 0.25 ug/mL biotinylated polyclonal goat antibody (BAF885, R&D Systems, Abingdon, United Kingdom) were added to each well, and plates left two hours at room temperature. Signal was amplified with Avidin-horseradisch-peroxidase (BD Pharmingen, Oxford, United Kingdom) and plates incubated during 30 minutes at RT. Finally, SureBlue Reserve TMB (KPL, Gaithersburg, United States of America) was added. Reactions were stopped by adding 100 μL HCl 1M. Absorbance was measured at 450 nm and results were expressed in percentage relative to normal plasma, using its serial dilution as standard curve. This ELISA was specific for human Gas6, with no cross-reactivity with human PS. Gas6 plasma level was measured in all patients at admission (Day 0), Day 3, Day 7 and the weekly until discharge from ICU or death. We used a commercial kit from R&D Systems (DY154, Abingdon, United Kingdom) to measure sAxl, following the instruction provided by the manufacturer. sAxl plasma level was expressed in ng/mL and measured in all patients only at admission (Day 0).

### Measurement of plasma levels of other circulating biomarkers

Other circulating biomarkers like CRP, PCT, and cytokines were measured on Day 0, as previously described [7].

### Statistical analysis

Gas6 and sAxl levels between survivors and non-survivors and between septic patients with sepsis and without shock were compared with the two-sample Wilcoxon-Mann-Whitney rank sum test and differences among proportions derived from categorical data were compared using the Fisher exact test. APACHE II and SOFA scores, CRP and PCT are expressed as median and range.

To study if Gas6 or sAxl level at admission predicted the ICU-mortality, we used Cox regression models in which Gas6 or sAxl were entered as continuous variables. We performed only an univariate analysis because the number of patients by subgroups was too small to realize a multivariate analysis.

A cut-off of plasma Gas6 concentration (249%) was defined as the value with the maximum of specificity and sensitivity to discriminate between survivors and non-survivors. A further analysis compared survival in patients having ≥ 249% vs. those having <249% Gas6 level. Log-rank test was used to compare the two groups.

To compare Gas6 evolution over time in deceased *vs*. surviving patients, we used linear mixed models, with random constants. We estimated the effect of survival, time and the interaction between time and survival on Gas6.

The association between Gas6 and the other variables measured was assessed with Spearman correlation coefficients.

Receiver operating characteristic (ROC) curve was applied to examine the performance of variables to predict intra-ICU mortality.

All *P* values were two-sided and considered statistically significant if the *P* value was less than or equal to 0.05.

Statistical analyses and graphics were performed using the statistical packages STATA (StataCorp LP, College Station, Texas, United States of America) and Prism Graphpad (Graphpad Software, La Jolla, California, United States of America).

## Results

### Patient characteristics

**Fig 1:**
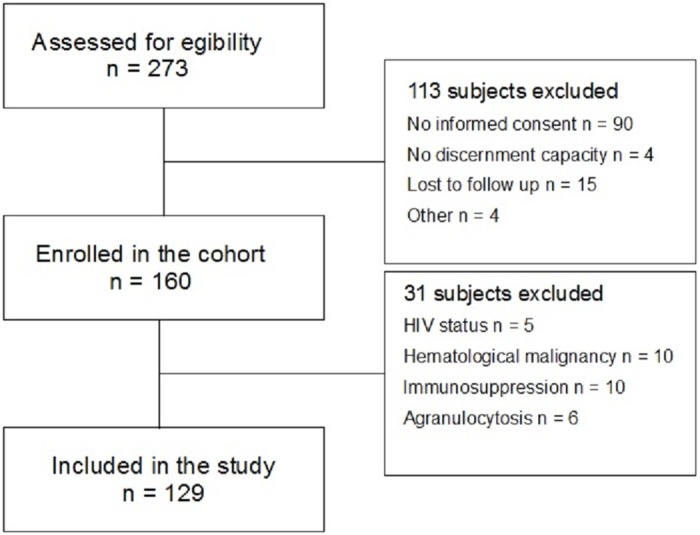
Flow diagram of patients included in the study.

A total of 273 patients were prospectively screened (Fig 1); of these, 113 (41%) were excluded (54 declined consent, 22 died before consent could be obtained, 14 were transferred to another hospital before being enrolled, 4 had no discernment capacity, 15 were lost to follow up because of transfer to another hospital or failure to collect blood samples or clinical data, 2 had no infection, 1 spoke Russian and 1 did not have blood draw for technical reasons). From the 160 enrolled in the cohort, 5 were excluded because of HIV status, 10 because of hematological malignancy, 10 because of immunosuppression, 6 because of agranulocytosis.

A total of 129 patients were included in the study. Patients’ characteristics and clinical parameters are listed in Table 1. There was a male predominance (61.2%) and a median age of 66 years (range 18–85). We reported 47 cases of sepsis without shock (36.4%) and 82 of sepsis with shock (63.6%). Ninety-three (72.1%) infections were community-acquired, 28 (21.7%) nosocomial and 8 (6.2%) health-care associated. The three main diagnoses at admission in the ICU were pneumonia (38.8%), peritonitis (24.8%) and fasciitis (10.1%) and the most common pathogens isolated from patients were *E*. *coli* (19.4%), *S*. *pneumoniae* (17.1%), *S*. *pyogenes* (7%). In 21.7% of the cases no pathogen was identified.

**Table 1:** Patient characteristics by survival status.

Patient characteristic	All Patients (n = 129)	Survivors (n = 104)	Non-survivors (n = 25)	P-value
Age, median years (range)	66 (18–85)	63 (18–85)	75 (55–85)	<0.001^1^
Sex, male	79	65 (62.5%)	14 (56.0%)	0.649^2^
Sepsis without shock	47	45 (43.3%)	2 (8.0%)	0.001^2^
Septic shock	82	59 (56.7%)	23 (92.0%)	0.001^2^
Cause of sepsis with/without shock				0.784^2^
Pneumonia	50	38 (36.5%)	12 (48.0%)	
Peritonitis	32	26 (25.0%)	6 (24.0%)	
Fasciitis	13	12 (11.5%)	1 (4.0%)	
Cholangitis	6	4 (3.8%)	2 (8.0%)	
Pyelonephritis	9	7 (6.7%)	2 (8.0%)	
Primary bacteremia	3	3 (2.9%)	0 (0.0%)	
Meningitis	4	4(3.8%)	0 (0.0%)	
Abscess	2	2 (1.9%)	0 (0.0%)	
Endocarditis	4	3 (2.9%)	1 (4.0%)	
Others	6	5 (4.8%)	1 (4.0%)	
Pathogens				0.711^2^
*E*. *coli*	25	21 (20.2%)	4 (16.0%)	
*S*. *pneumoniae*	22	18 (17.3%)	4 (16.0%)	
*S*. *pyogenes*	9	8 (7.7%)	1 (4.0%)	
*P*. *aeruginosa*	7	6 (5.8%)	1 (4.0%)	
*S*. *aureus* (MRSA)	9 (1)	7 (1) (6.7%)	2 (0) (8.0%)	
Other pathogen isolated	29	25 (24.0%)	4 (16.0%)	
No pathogen identified	28	19 (18.3%)	9 (36.0%)	
Origin				0.214^2^
Community-acquired	93	76 (73.1%)	17 (68.0%)	
Nosocomial	28	20 (19.2%)	8 (32.0%)	
Health-care associated	8	8 (7.7%)	0	
Comorbidities				
Diabetes (with insulin therapy)	26 (7)	20 (6) (19.2%)	6 (1) (24.0%)	0.586^2^
COPD	20	16 (15.4%)	4 (16.0.6%)	1.000^2^
Coronary artery disease	22	19 (18.3%)	3 (12.0%)	0.565^2^
McCabe classification				0.042^2^
Non-fatal	77	67 (64.4%)	10 (40%)	
Ultimately fatal (< 5 years)	39	29 (27.9%)	10 (40%)	
Rapidly fatal (< 6 months)	13	8 (7.7%)	5 (20%)	
APACHE II, median (range)	28 (13–46)	27 (13–46)	33 (22–45)	0.004^1^
SOFA, median (range)	11 (5–20)	10 (5–20)	13 (6–19)	0.010^1^
CRP, median (range)	257 (4–558)	262 (4–558)	225 (70–538)	0.383^1^
PCT, median (range)	21.4 (0.1–201)	21.3 (0.1–201)	24.6 (1.7–201)	0.328^1^

^1^ Mann-Whitney test.

^2^ Fisher’s exact test.

APACHE II = Acute Physiology and Chronic Health Evaluation II, COPD = Chronic Obstructive Pulmonary Disease, CRP = C-reactive protein, MRSA = methicillin resistant *S*. *aureus*, PCT = Procalcitonin, SOFA = Sequential Organ Failure Assessment.

With respect to patient co-morbidities, 20.2% had diabetes but only a quarter of them required insulin, 15.5% suffered from COPD and 17.1% had coronary artery disease. The in-ICU mortality represented 19.4%, the 28-day mortality 22%, the in-hospital mortality 26% of the cohort's population. The vast majority of non-survivors suffered from septic shock (23 out of 25).

The severity scores used were APACHE II with median value of 28 points (range 13–46) and SOFA with median value of 11 points (range 5–20). Ten percent had McCabe classification predicting rapidly fatal outcome. The median value for CRP was 257 mg/L (range 4–558) and for PCT 21.4 μg/L (range 0.1–201). Age (*P* <0.001), septic shock (*P* = 0.001), APACHE II score (*P* = 0.004) and SOFA score (*P* = 0.010) were higher in non-survivors. McCabe classification was higher in survivors (*P* = 0.042). The etiological agent, the final diagnosis, CRP and PCT levels and the presence of diabetes, COPD or coronary artery disease were not statistically different between survivors and non-survivors.

### Plasma levels of Gas6 and sAxl

**Fig 2:**
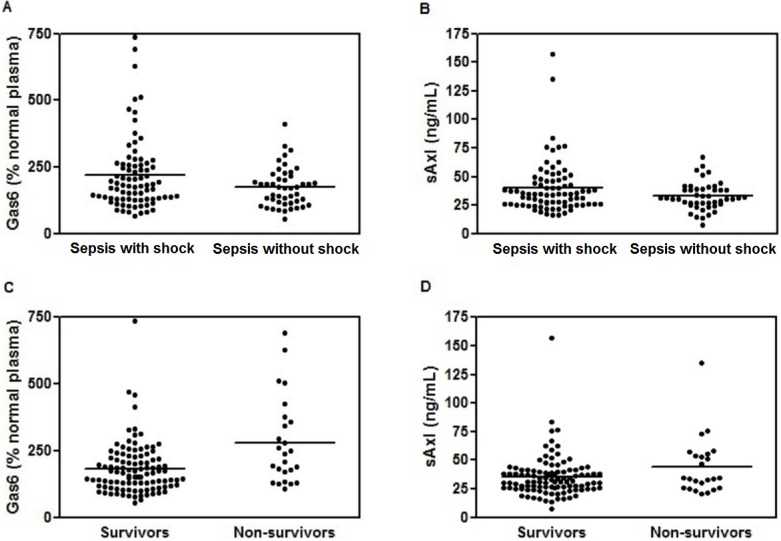
Gas6 and soluble Axl (sAxl) levels. **(A)** Gas6 plasma levels at admission in septic patients with shock (median 180.5, IQR 130) and without shock (median 173, IQR 104) (*P* > 0.05, Wilcoxon rank-sum (Mann-Whitney) test), **(B)** sAxl plasma levels at admission in septic patients with shock (median 35, IQR 20.5) and septic patients without shock (median 31, IQR 13) (*P* > 0.05, Wilcoxon rank-sum (Mann-Whitney) test), **(C)** Gas6 plasma levels at admission in survivors (median 167%, IQR 106) and non-survivors (median 238%, IQR 217) (*P* = 0.003, Wilcoxon rank-sum (Mann-Whitney) test), **(D)** sAxl plasma levels at admission in survivors (median 31, IQR 14.5) and non-survivors (median 34.5, IQR 29) (*P* > 0.05, Wilcoxon rank-sum (Mann-Whitney) test).

Median plasma Gas6 and sAxl levels at admission were comparable in septic patients with and without shock (Fig 2A and 2B, *P* > 0.05). There were no significant differences of Gas6 and sAxl levels between bacteriemic and non-bacteriemic patients and between Gram-negative and Gram-positive infections.

However, Gas6 levels at admission were higher in non-survivors (median 238%, IQR 217) than in survivors (median 167%, IQR 106) (Fig 2C, n = 129, *P* = 0.003). The relative risk of death during the ICU stay was multiplied by 1.0037 for each supplementary percentage of plasma Gas6 level at admission (Cox regression, 95% CI: 1.0014–1.0059, *P* = 0.001). An augmentation of 50% of Gas6 level at admission increased by 20% in the relative risk of death during the ICU stay (Hazard Ratio: 1.20, 95% CI: 1.07–1.34%).

Similarly, non-survivors exhibited a higher sAxl plasma levels than survivors (median 34.5 ng/ml, IQR 29 *vs* median 31 ng/ml, IQR 14.5 respectively), but this was not statistically significant (*P* = 0.163, Fig 2D).

**Fig 3:**
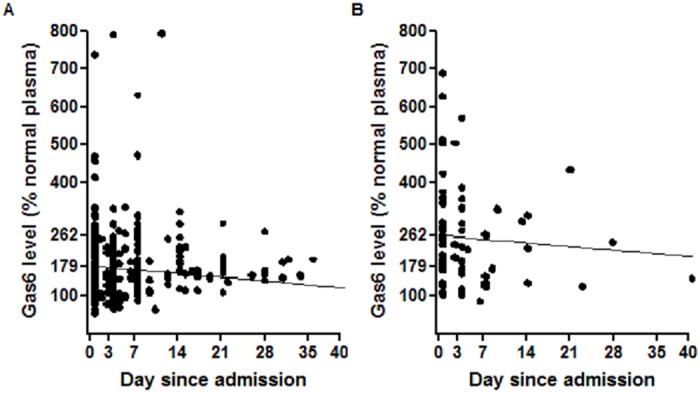
Evolution of Gas6 levels during ICU stay. The scheme is based on a linear mixed model without interaction between time in ICU and survival. Round represent the observed measures in survivors (a) and non-survivors (b), respectively. Lines represent the model-fitted values.

The linear mixed model to study Gas6 evolution during the ICU stay demonstrated that the interaction between survival and time was not statistically significant. This analysis suggests that difference in Gas6 level between survivors and non-survivors remained constant over time. Independently from time, non-survivors had a mean level of Gas6 83% higher than survivors. We therefore proposed a second model that excludes the time-survival interaction (Fig 3). According to this last model, non-survivors exhibited a mean Gas6 level of 262% versus 179% in survivors at admission (*P* for the difference <0.001). Gas6 then decreased by 1.36% (*P* = 0.010) each day in both groups. Whatever model we used, the Gas6 curve remained more elevated in non-survivors than in survivors.

As the number of non-survivors diminished rapidly after D0, we focused our next analysis on results from the first blood sample.

### Correlation of Gas6 with biomarkers of inflammation and severity scores

Gas6 levels at admission correlated positively with plasma levels of IL-6, IL-8, IL-10 and sAxl. Correlation with IL-1beta and TNF-alpha was not significant. PCT correlated with Gas6, while CRP, SOFA and APACHEII scores did not (Table 2).

**Table 2:** Correlation between the plasma levels of Gas6 and those of other biomarkers.

Biomarker	rho	P
sAxl	0.33	<0.001
IL-10	0.31	<0.001
IL-8	0.31	<0.001
IL-6	0.26	0.004
PCT	0.23	0.009
TNF-alpha	0.14	0.113
SOFA	0.11	0.212
IL-1	0.06	0.507
APACHE II	0.04	0.212
CRP	-0.10	0.270

APACHE II = Acute Physiology and Chronic Health Evaluation II, CRP = C-reactive protein, IL = Interleukin, PCT = Procalcitonin, SOFA = Sequential Organ Failure Assessment, TNF = Tumor necrosis factor.

### Biomarkers and severity scores as predictors of intra-ICU mortality

Except Gas6, the only biomarkers found to be differently expressed between survivors and non-survivors were IL-8 (*P* = 0.003) and IL-10 (*P* = 0.004). Both SOFA and APACHE II scores predicted mortality during ICU stay (*P* = 0.011 and 0.004, respectively).

**Fig 4:**
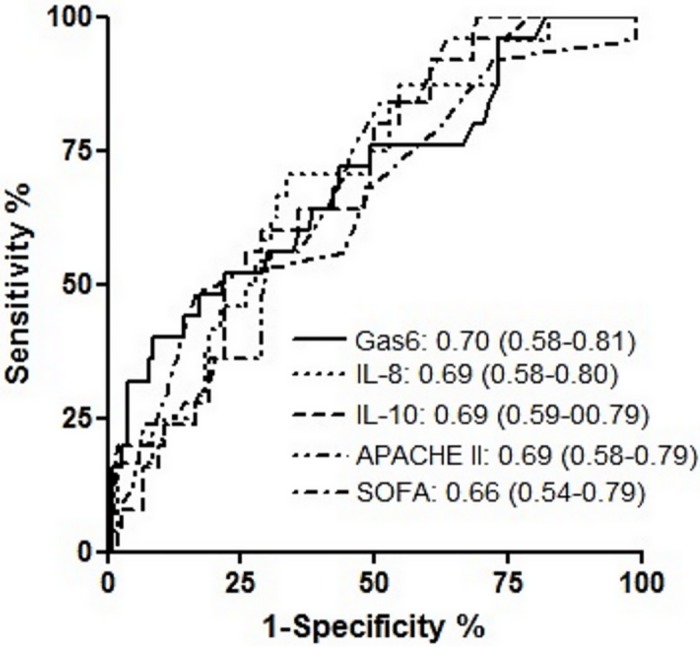
Receiver operator characteristic curve analysis of blood parameters for death in the ICU. The value of the Area Under the Curve for each blood parameter and the 95% confidence intervals are listed on the bottom right of the figure. APACHE II = Acute Physiology and Chronic Health Evaluation II, IL = Interleukin, SOFA = Sequential Organ Failure Assessment.

Table 3 shows the area under the curve (AUC) of the different markers and Fig 4 illustrates the ROC curves of the blood parameters and clinical scores which are statistically different between survivors and non-survivors. Based on the ROC curve area, Gas6 has, albeit modestly, the highest diagnostic efficacy of intra-ICU mortality.

**Table 3:** Performance of different biomarkers to predict mortality based on ROC curve area.

Biomarker	ROC area	SE	95%CI
Gas6	0.695	0.061	0.576–0.814
IL-8	0.693	0.056	0.584–0.803
IL-10	0.688	0.052	0.586–0.790
APACHE II	0.686	0.052	0.584–0.788
SOFA	0.664	0.063	0.540–0.788
TNF-alpha	0.624	0.059	0.509–0.739
IL-6	0.623	0.064	0.498–0.748
sAxl	0.592	0.069	0.458–0.727
IL-1beta	0.570	0.071	0.431–0.708
PCT	0.563	0.058	0.449–0.677
CRP	0.556	0.062	0.434–0.678

SE = Standard Error, 95% CI = 95% Confidence Interval, APACHE II = Acute Physiology and Chronic Health Evaluation II, CRP = C-reactive protein, IL = Interleukin, PCT = Procalcitonin, SOFA = Sequential Organ Failure Assessment, TNF = Tumor necrosis factor.

**Fig 5:**
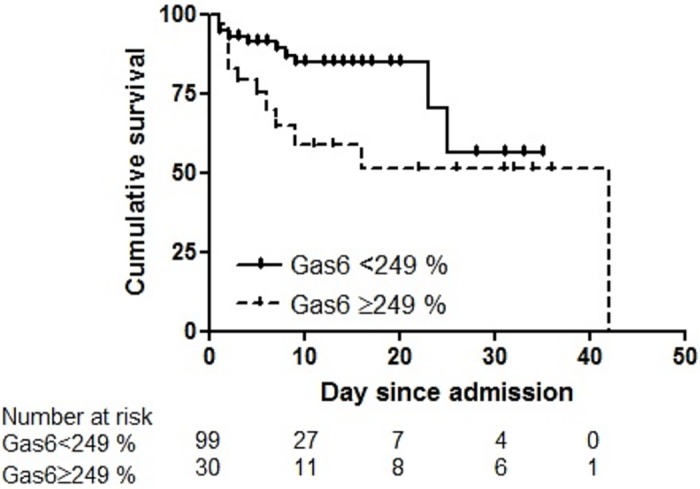
Survival curves of patients with sepsis with or without shock. Using Kaplan-Meier curves, stratified by Gas6 measurement at admission with a cut-off value of 249% (relative to Gas6 concentration in normal plasma) (*P* = 0.026, Log-rank test).

Considering 249% as a cut-off value, which was result driven, Gas6 measurement had a specificity of 82.7% (95% CI: 74%-89%) and a sensitivity of 48% (95% CI: 28%-69%) with a positive likelihood ratio of 2.77 for predicting mortality. Positive and negative predictive values for mortality were 40% and 87%, respectively. A log-rank test showed that there was a significant difference (*P* = 0.026) between survival curves in the two groups (Fig 5).

## Discussion

Our data suggest that Gas6, measured within 24 hours of ICU admission, may help to predict the risk of mortality in septic patients with and without shock. Previous work showed that high plasma Gas6 level correlates with the degree of organ dysfunction. Endogenous Gas6 has a homeostatic role by acting on macrophages and plays a beneficial role in murine models of endotoxemia [29]. Gas6 attenuates the systemic inflammatory response in the early phase of sepsis. Recently, it has been shown that recombinant Gas6 rescues mice from mortality following cecal ligation and puncture and reduces both plasma cytokine levels and cellular infiltration in the lungs [29]. However, Gas6 might also contribute to the hypo-inflammatory response in a later phase of sepsis and participate to a compensatory anti-inflammatory response syndrome that might results in immunoparalysis with inability to clear the original pathogen invasion or predisposes to secondary infection [40].

The present study proposes that Gas6, measured within 24 hours of ICU admission, may be used as a biomarker to identify septic patients at highest risk of death. Nevertheless, Gas6 level was not different between septic patients with or without shock. The strength of the present study is that included patients are representative of the population usually admitted to a medico-surgical ICU. Clinical scores (SOFA and APACHEII) predicted mortality during ICU stay. Because Gas6 is a modulator of innate immunity, patients with HIV status, hematological malignancies, immunosuppression or agranulocytosis were excluded. Gas6 performed better than PCT and CRP, both broadly used to diagnose infection but, because of their poor accuracy in this specific indication, should not be used to predict disease outcome. A major difference between Gas6 and currently used predictors of sepsis evolution, such as cytokines, is the absence of time-survival interaction. Indeed, Gas6 level remained more elevated in non-survivors than in survivors during the 28-day observation time. Although Gas6 level slightly decreased each day both in survivors and non-survivors, this diminution was not statistically significant. This biomarker behavior contrasts with that of pro-inflammatory cytokines like TNF-alpha, IL-1, IL-6, which are characterized by their transient increase in plasma, rendering the interpretation of their plasma level difficult in patients in whom the time between the onset of sepsis and the blood draw cannot be standardized [41]. On contrary, because Gas6 level did not follow a significant time course, it would not be a suitable biomarker to appreciate the patient’s response to therapy. Finally, considering Gas6 plasma level of 249% as a cut-off value, Gas6 measurement had a negative predictive value for mortality of 87%. This last finding may allow to identify potential survivors.

Our study has also several limitations. First, since 113 out of 273 (41%) patients were excluded mainly because informed consent could not be obtained within 24 hours (90 patients, 33%), a selection bias cannot be excluded. Second, owing to organizational constraints, patient recruitment was restricted to periods when investigators worked as attending physicians. Third, since the present study is a cohort-based study of patients with a definite diagnosis of sepsis at the time of ICU admission, patients without sepsis were not included. Fourth, owing the relatively small number of patients, these results require further validation in a larger and multi-centric cohort of patients with sepsis, which could, furthermore, allow extensive model making.

Overall, our data suggest that plasma Gas6 level measurement could be useful to stratify patients with sepsis. After initial resuscitation, Gas6 level determined within 24 hours of ICU admission may be used with clinical and multi-marker panel, of both pro- and anti-inflammatory markers [5,8], to select patients at high risk of death and to reduce the number of those susceptible to receive novel and expensive adjunctive therapies.

## Conclusion

We conclude that a single measurement of plasma Gas6 level within 24 hours of ICU admission stratifies septic patients with or without shock according to risk of death. If our findings are confirmed by further studies, this promising biomarker may help critical care physicians to tailor therapy to individual patients according to risk of death.
